# Identification and sequence analysis of prolactin receptor and its differential expression profile at various developmental stages in striped hamsters

**DOI:** 10.1590/1414-431X202010274

**Published:** 2021-03-15

**Authors:** Huiliang Xue, Jinhui Xu, Ming Wu, Lei Chen, Laixiang Xu

**Affiliations:** 1 College of Life Sciences, Qufu Normal University, Qufu, Shandong, China

**Keywords:** PRLR, Striped hamster, Developmental stages

## Abstract

Prolactin (PRL) plays critical roles in regulation of biological functions with the binding of specific prolactin receptor (PRLR). Revealing the expression patterns of PRLR at different developmental stages is beneficial to better understand the role of PRL and its mechanism of action in striped hamsters. In this study, the cDNA sequence of PRLR (2866-base-pairs) was harvested from the pituitary of mature female striped hamsters (*Cricetulus barabensis*) that contains an 834-base-pair 5′-untranslated region (1-834 bp), a 1848-base-pair open reading frame (835-2682 bp), and a 184-base-pair 3′-untranslated region (2683-2866). The 1848-base-pair open reading frame encodes a mature prolactin-binding protein of 592 amino acids. In the mature PRLR, two prolactin-binding motifs, 12 cysteines, and five potential Asn-linked glycosylation sites were detected. Our results showed that the PRLR mRNA quantity in the hypothalamus, pituitary, ovaries, or testis was developmental-stage-dependent, with the highest level at sub-adult stage and the lowest level at old stage. We also found that PRLR mRNAs were highest in pituitary, medium level in hypothalamus, and lowest in ovaries or testis. PRLR mRNAs were significantly higher in males than in females, except in the hypothalamus and pituitary from 7-week-old striped hamsters. Moreover, the PRLR mRNAs in the hypothalamus, pituitary, and ovaries or testis were positively correlated with the expression levels of GnRH in the hypothalamus. These results indicated that the PRLR has conserved domain in striped hamster, but also possesses specific character. PRLR has multiple biological functions including positively regulating reproduction in the striped hamster.

## Introduction

The striped hamster (*Cricetulus barabensis*) is one of main rodent pests in northern China farmland that has high reproductive capacity. It generally reproduces three times a year, and the litter size is of four to nine for every parity (1). The high reproductive activity is one of main reasons leading to the population fluctuation (2). In general, rodents play an important role in maintaining the ecological balance, however, when there is an outbreak, rodents cause enormous damage to agricultural production (3).

Prolactin (PRL) is a single-chain peptide hormone that is mainly synthesized and secreted by prolactin cells in the anterior pituitary, and acts on breast development, lactation maintenance, reproduction (4), as well as on growth and development, metabolism, immuno-regulation, energy balance, and behavior (5,6). PRL acts by binding to a specific membrane prolactin receptor (PRLR) (7,8), which belongs to the class I cytokine receptor superfamily (9). PRLR is a single membrane-bound protein expressed in the hypothalamus, pituitary, breast, ovary, testis, liver, and many other tissues of mammalians (10). The circulating levels of PRL are highly correlated with the levels of PRLR mRNA in both the pituitary gland and hypothalamus, which indicates that PRL positively regulates the PRLR expression in the hypothalamus (11) and biological actions of PRL are mediated by PRLR simultaneously.

The expression levels of PRLR are much higher in the hypothalamus and pituitary gland than in other tissues (12). Therefore, PRLR participates in the biological function of PRL, and its specific function and expression level are tissue-dependent. However, the mechanism involved in PRL binding to PRLR with the transferring of hormonal signal inside the cell remains unknown. The study on primary structure and expression patterns of PRLR may facilitate the understanding of the PRL mechanism of action. Since the action of PRL is initiated by its specific receptor on the cell surface (13), a detailed characterization of PRLR is necessary to further understand the PRL action mechanism. The cysteine residues and N-linked glycosylation sites are important to build specific constructs and execute specific activities for PRLR. Whether conserved cysteine residues and N-linked glycosylation sites also exist in the striped hamster PRLR is still not well understood.

In humans, prolactin is secreted daily with the highest level during sleep and the lowest level during waking hours (14,15), which may be regulated by the suprachiasmatic nuclei of the hypothalamus (16). The transcription of PRLR in the ovaries is a complicated process regulated by developmental stage and hormones (17). The expression of PRLR in the hypothalamus is increased by prolactin in serum (18). Estrogens can also increase the expression level of PRLR in the brain (19). Therefore, the PRLR expression profile may vary with the different developmental stages.

To date, the PRLR sequence character and the PRLR mRNA changes in various developmental stages in the striped hamster are little understood. Therefore, our present study used the mature female striped hamster as an experimental model to reveal the cDNA sequence and the deduced amino acid sequence of PRLR and examine the expression patterns of PRLR at different developmental stages in striped hamsters.

## Material and Methods

### Preparation of animals and tissues

The striped hamsters (*Cricetulus barabensis*) used in this study were captured by live-trap method using iron cages in the fields of Puwang Village, Yinan County, Linyi City of Shandong Province, China. Six mature female striped hamsters were used to make cDNA libraries. Eighteen male and female animals (six hamsters of 7 weeks (18±1 g), 13 weeks (24±1 g), and 1.5 years old (30±1 g)) were used to examine the PRLR expression profile. The weight among animals in the same developmental stage and same sex deviated no more than 1 gram. Animals were treated in accordance with the Animal Ethics Committee of Qufu Normal University.

The selected animals were maintained on a 12-h light/dark cycle schedule (lights on at 0400 hour) at Qufu Normal University (China) in the Animal Experiment Center at 20°C conditions for one week. The 7-week and 1.5-year old animals were then killed by CO_2_ asphyxiation, and were dissected to collect the hypothalamus, pituitary, and ovaries or testes, which were stored at -80°C. For the 13-week hamsters, from 5 to 6 pm after one week, the estrous cycle of females was examined by vaginal smears and the estrus period of males was judged by estrous behavior. When the animals were in estrous period, they were immediately killed by CO_2_ asphyxiation, and dissected to collect the hypothalamus, pituitary, and ovaries or testes tissues, which were stored at -80°C. All the experiments were approved by the Animal Ethics Committee of Qufu Normal University.

### Total RNA extraction

Total RNA of the hypothalamus, pituitary, and ovaries or testis of the selected animals were extracted using RNA extraction reagent (TaKaRa, Japan). The purity and the concentration of total RNA were assessed by the ratio of D260 to D280 using an ultraviolet spectrophotometer (Eppendorf, Germany). The integrity of total RNA was also examined by agarose gel electrophoresis (AGE). Subsequently, all high-quality total RNA from the sampled tissues were reverse transcribed to cDNA using reverse transcriptase XL AMV (TaKaRa) with an Oligo deoxy thymidylate primer and random primers. All synthesized cDNA was stored at -20°C

### Preparation and screening of cDNA libraries

The oligo (dT) and random primers were used to construct cDNA libraries from the pituitary glands of the six mature females. A sense primer (P1, 5′-GTCCAGACTCGCTGCAAGCC-3′) and an antisense primer (P2, 5′-AGATGCAGGTCATCATGCTA-3′) were designed based on the conserved sequences of the golden hamster (XM_021231782.1), *Mus musculus* (NM_011169.5), and *Rattus norvegicus* (NM_001034111.1) PRLR and were used for the amplification of a cDNA fragment from the striped hamsters PRLR mRNA. A 193-bp fragment of PRLR cDNA amplified by RT-PCR using P1 and P2 primers was used as the probe to screen the cDNA libraries. Hybridization was performed in 20% formamide at 42°C and the filters were washed in 1×SSC (0.15 M NaCl, 0.015 M sodium citrate, pH 7) at 42°C.

### Gene characterization

Similarity assessment and homology searches of the nucleotides and amino acids of the PRLR from the striped hamster were analyzed by NCBI BLAST (http://www.ncbi.nlm.nih.gov/blast) (20). SignalP 4.0 (http://www.cbs.dtu.dk/services/SignalP/) (21) and Tmpred (http://www.ch.embnet.org/software/TMPRED_form.html/) (21) were used to detect the protein signal peptide and the transmembrane structures, respectively.

### Real-time fluorescence quantitative polymerase chain reaction

Based on the cloned nucleotide sequences of PRLR from the striped hamsters, primers of real-time quantitative polymerase chain reaction (RT-qPCR) of PRLR were designed using Beacon Designer 7.0 software (http://www.premierbiosoft.com). The primers of PRLR were F1 (5′- GCATCTTTCCACCAGTTCC-3′) and R1 (5′-TTGGCATCCTAAGGCAGT-3′). The primers of β-actin (as a reference gene) were F2 (5′-GAGACCTTCAACACCCCAGC-3′) and R2 (5′-ATGTCACGCACGATTTCCC-3′). RT-qPCR was performed using an Agilent Stratagene Mx3000P detector (USA) with the Brilliant II SYBR Green qPCR Master mix (TaKaRa). Three repeats for each sample were performed during every RT-qPCR test.

The volume of reaction system for RT-qPCR was 20 µL, including 10 μL SYBR Green, 0.4 μL forward primer and reverse primer (10 μM), respectively, 2 μL cDNA template, 0.3 μL ROX, and 6.9 μL DEPC H_2_O. The reaction procedure was 10 min at 94°C for enzyme activation followed by 40 cycles of 1 min at 94°C, 1 min at 56°C, and 1 min at 72°C.

The fluorescence signal was collected during every PCR cycle at the renaturation step and was positively correlated with the quantity of the product. The specificity of the product was confirmed using melting curve analysis with a single peak per unique amplification, and the integrity of the product was examined by 2.5 % AGE. The amplification efficiency for the specific primers was also tested using the standard curve, which should be from 90 to 110% (22). The expression level of PRLR mRNA is reported with the 2^-ΔΔCT^ method (normalization to β-actin) (23).

### Statistical analysis

The data are reported as means±SE. Statistical analysis was conducted using the independent sample *t*-test between two groups and the single-factor analysis of variance (ANOVA) among more than two groups. The differences were considered to be significant when P<0.05 and highly significant when P<0.01. Pearson's correlations were determined for PRLR relative expression levels in the hypothalamus with GnRH mRNA quantity in the hypothalamus. Data were analyzed using SPSS 17.0 (IBM, USA).

## Results

### Character of the cDNA sequence and the deduced amino acid sequence of PRLR

**Figure 1:**
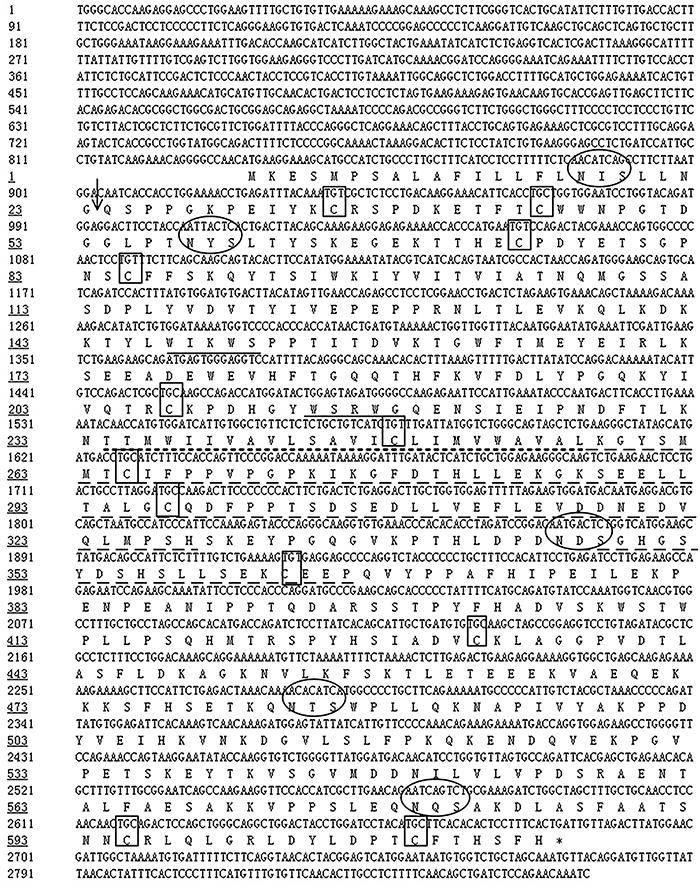
Prolactin receptor (PRLR) cDNA sequence and inferred amino acid sequence of *Cricetulus barabensis* (n=6). Top sequence is the nucleotide sequence and bottom sequence indicates the deduced amino acid residues. The nucleotide sequence deduced to amino acid residues was open reading frame, including 1848-base-pairs (835-2682 bp). The nucleotide sequence 1-834 bp is in the 5′-untranslated region, and the nucleotide sequence 2683-2866 bp is in the 3′-untranslated region. The signal peptide (1-23 amino acid residues) is marked by an arrow, and the transmembrane domain (234-257 amino acid residues) is underlined by short dashed line. Two conserved amino acid residue sequences at the intracellular domain are underlined by long dashed lines. Two prolactin-binding motifs (WIKWS and WSRWG) at the extracellular domain are marked by solid line. The cysteines are boxed and the Asn-linked glycosylation sites are circled.

The cDNA sequence of PRLR (2866-base-pairs) in the pituitary of the mature females is shown in Figure 1, including an 834-base-pair 5′-untranslated region (1-834 bp), a 1848-base-pair open reading frame (835-2682 bp), and a 184-base-pair 3′-untranslated region (2683-2866). The 1848-base-pair open reading frame encoded a peptide of 615 amino acids, including a signal peptide of 23 amino acids and a mature prolactin-binding protein of 592 amino acids. The mature prolactin-binding protein contained the extracellular prolactin-binding region of 210 residues at amino-terminal, the transmembrane region of 24 residues, and the intracellular domain of 358 residues at carboxyl-terminal. In the mature prolactin-binding protein, 12 cysteines were detected, including 5 cysteines in the extracellular region, 1 cysteine in the transmembrane region, and 6 cysteines in the intracellular region. Five potential Asn-linked glycosylation sites were also detected, including 1 site in the signal peptide and extracellular region, respectively, and 3 sites in the intracellular domain. At the extracellular domain, two prolactin-binding motifs (WIKWS and WSRWG) were detected and two conserved parts (258-328 and 347-366 amino acids) were found in the intracellular domain.

### Expression profiles of PRLR among different developmental stages

**Figure 2:**
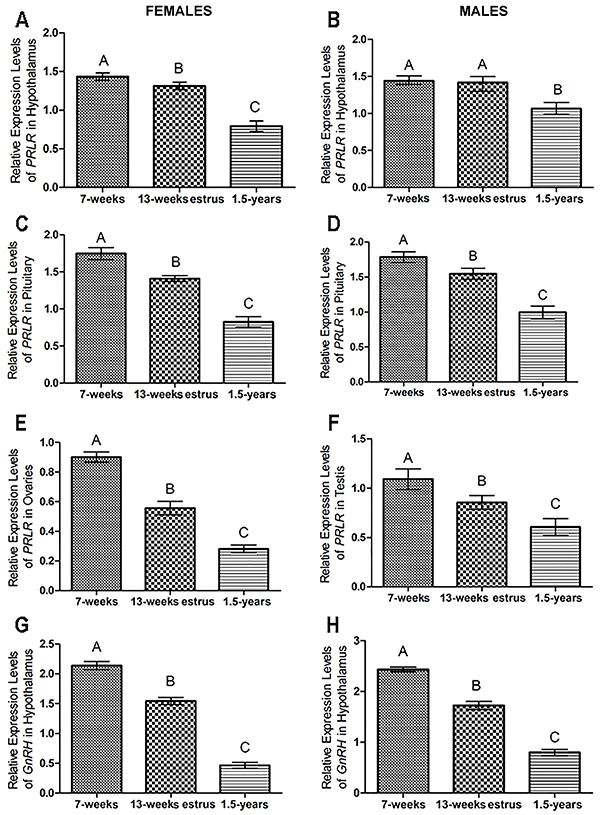
Relative expression levels of prolactin receptor (PRLR) mRNA in the hypothalamus, pituitary, and ovaries or testis, and GnRH mRNA in the hypothalamus of the striped hamster at different developmental stages (7 weeks, n=6; 13 weeks, n=6, 1.5 years, n=6) for (**A**, **C**, **E**, **G**) females and (**B**, **D**, **F**, **H**) males. The data are reported as means±SE. Different upper-case letters indicate significant differences (P<0.01). (ANOVA).

The expression levels of PRLR in the hypothalamus, pituitary, and ovaries or testis and that of GnRH in female or male animals were all developmental-stage-dependent. The highest expression levels of PRLR and GnRH were in 7-week-old animals, and the lowest in 1.5-year-old males and females. Medium expression levels of PRLR and GnRH were detected at 13-week estrous period. The expression levels of PRLR and GnRH from different developmental stages showed significant differences except for the difference of PRLR expression levels in the hypothalamus between the 7- and 13-week-old males (Figure 2).

### Expression profiles of PRLR among different tissues

**Figure 3:**
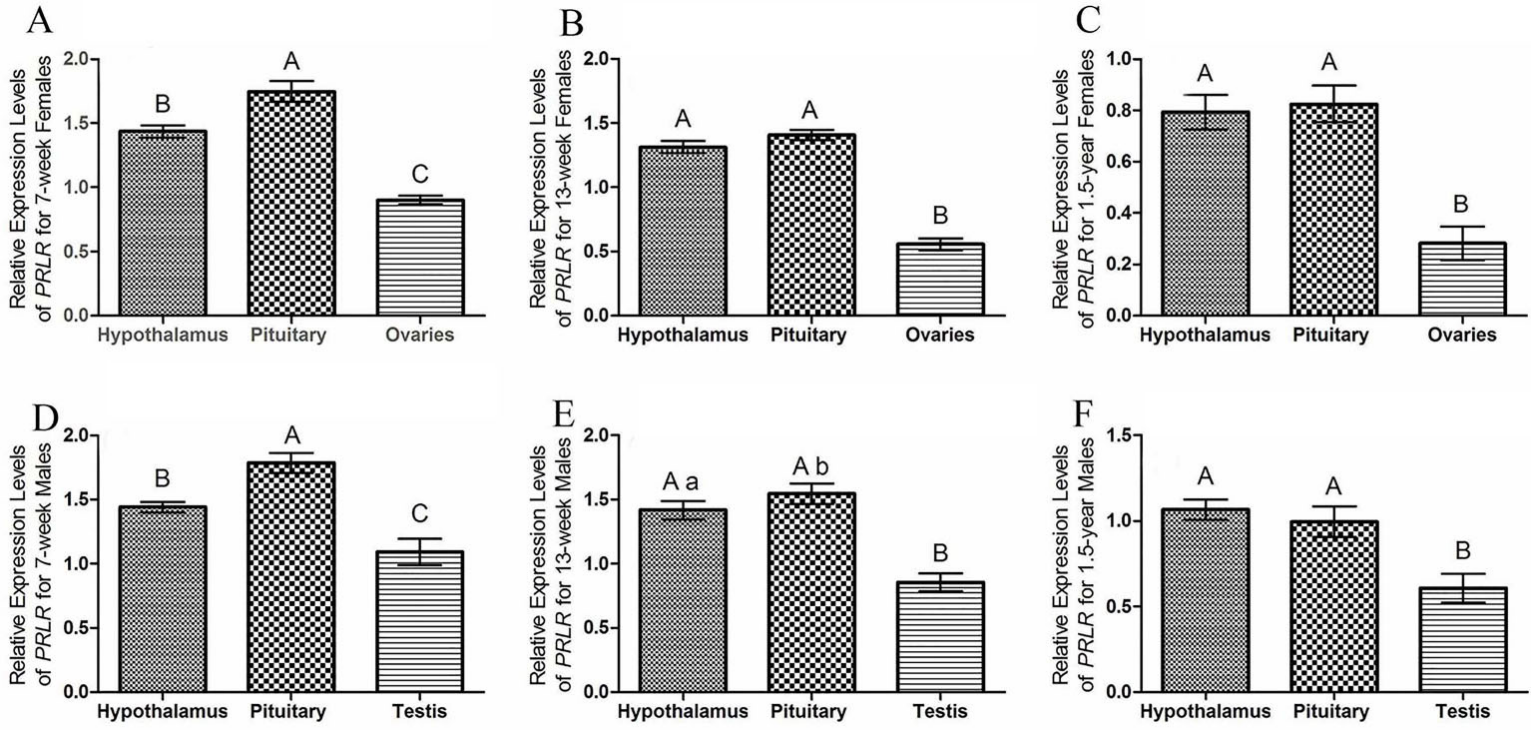
Relative expression levels of PRLR mRNA among the hypothalamus, pituitary, and ovaries or testis in the striped hamster at different developmental stages (7 weeks, n=6; 13 weeks, n=6, 1.5 years, n=6). Relative PRLR mRNA of the hypothalamus, pituitary, and ovaries from (**A**) 7-week, (**B**) 13-week estrous, and (**C**) 1.5-year female striped hamsters. Relative PRLR mRNA of the hypothalamus, pituitary, and testis from (**D**) 7-week, (**E**) 13-week estrous, and (**F**) 1.5-year male striped hamsters. The data are reported as means±SE. Different lower-case letters (P<0.05) and different upper-case letters (P<0.01) indicate significant differences. (ANOVA).

For 7-week-old females and males, expression of PRLR was significantly higher in pituitary and significant lower in ovaries or testis (P<0.01). There was medium expression of PRLR in hypothalamus for the 7-week-old females and males (Figure 3A and D). For 13-week-old estrous females and males, the expression levels of PRLR in hypothalamus and pituitary were significantly higher than in ovaries or testis (P<0.01). Significantly higher expression of PRLR (P<0.05) was found in pituitary than in hypothalamus in 13-week-old estrous males (Figure 3B and E). For 1.5-year-old females and males, the PRLR expression was significant higher in pituitary and hypothalamus than that in ovaries or testis (Figure 3C and F). The results showed that the expression of PRLR was tissue-dependent in the striped hamsters.

### Expression profiles of PRLR between males and females

**Figure 4:**
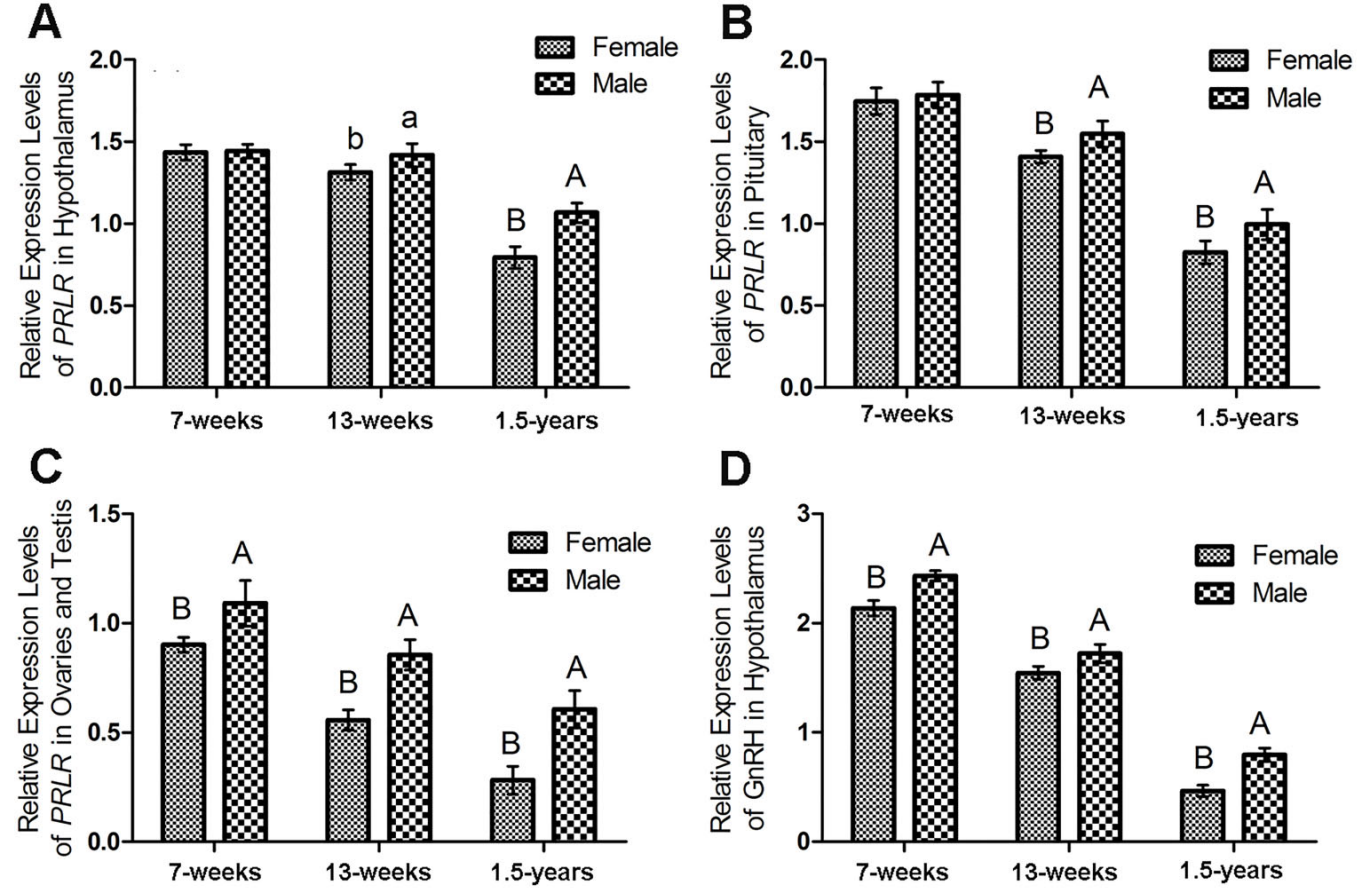
Relative expression levels of prolactin receptor (PRLR) mRNA between female and male striped hamsters (7 weeks, n=6; 13 weeks, n=6, 1.5 years, n=6) in (**A**) hypothalamus, (**B**) pituitary, (**C**) female ovaries and male testis, and (**D**) GnRH mRNA in the hypothalamus. The data are reported as means±SE. Different lower-case letters (P<0.05) and different upper-case letters (P<0.01) indicate significant differences (*t*-test).

For 7-week-old animals, no significant differences of PRLR mRNA were found in hypothalamus and pituitary between females and males (Figure 4A and B). Significant differences of PRLR mRNA in hypothalamus were found between females and males at 13-week-estrous (P<0.05) and 1.5-years (P<0.01), respectively (Figure 4A). Significantly higher PRLR mRNA was found in the pituitary of males than that of females at 13-week-estrous and 1.5-years (Figure 4B). PRLR mRNA was significantly higher in male testis than that in female ovaries (P<0.01) (Figure 4C) and the GnRH mRNA was significant higher in male hypothalamus than that in female hypothalamus (P<0.01) (Figure 4D) in 7- and 13-week-old, and 1.5-year-old striped hamsters.

### Correlation between expression levels of PRLR and GnRH in the hypothalamus

**Figure 5:**
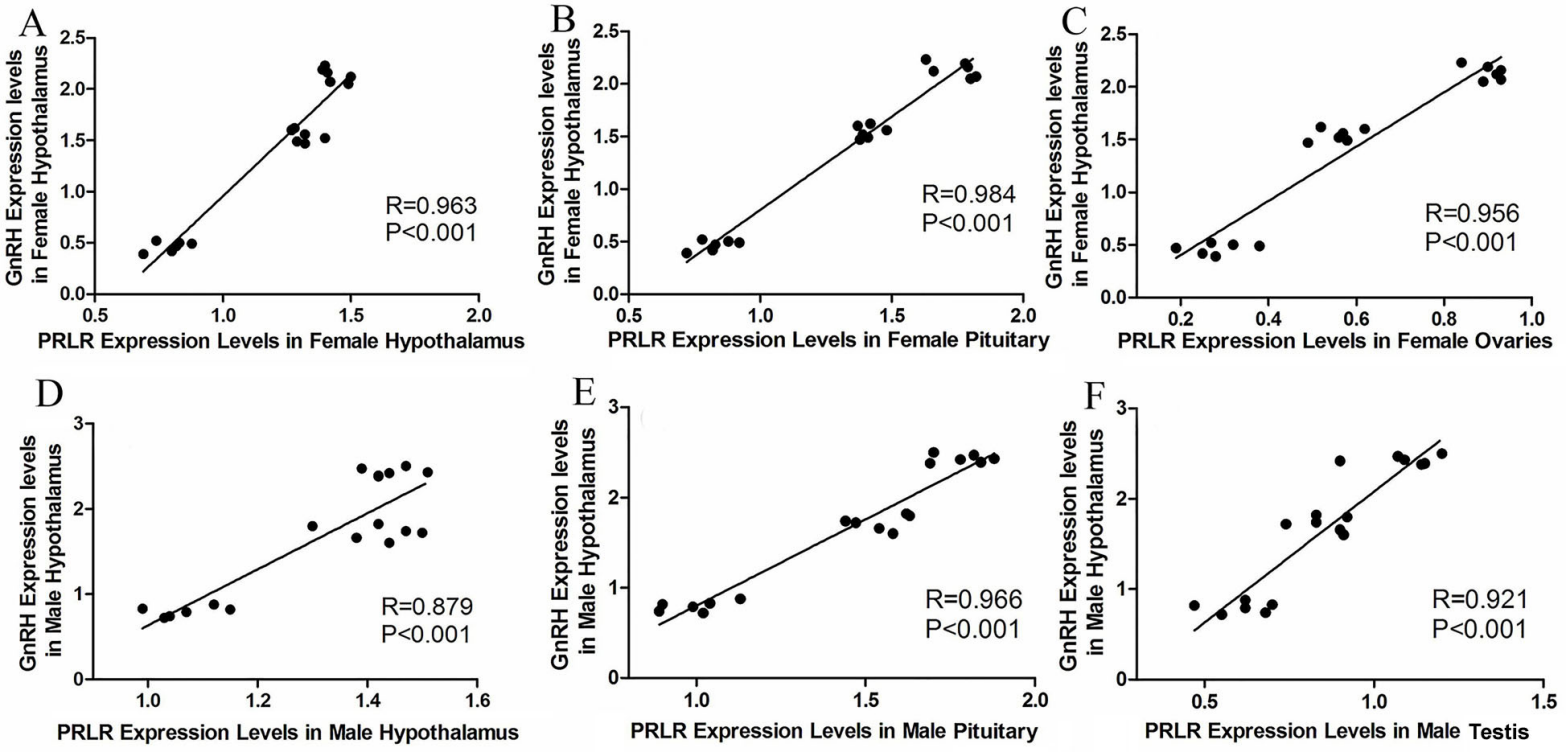
Correlations of the relative expression of prolactin receptor (PRLR) in the hypothalamus, pituitary, and ovaries (**A**-**C**) with that of GnRH in the hypothalamus of female striped hamsters. Correlations of PRLR mRNA in the hypothalamus, pituitary, and testis (**D**-**F**) with that of GnRH mRNA in the hypothalamus of male striped hamsters.

The relative expression levels of PRLR in the hypothalamus, pituitary, and ovaries or testis were positively correlated with the expression levels of GnRH in the hypothalamus of the striped hamster (Figure 5).

## Discussion

In this study, we found that the PRLR mRNA levels in the hypothalamus, pituitary, ovaries or testis were developmental-stage-dependent with the highest level at sub-adult stage and lowest level at old stage. PRLR mRNA levels were highest in pituitary, medium level in hypothalamus, and lowest in ovaries or testis. PRLR mRNA was significantly higher in males than in females, except in the hypothalamus and pituitary from 7-week-old striped hamsters. Moreover, the PRLR mRNA levels in the hypothalamus, pituitary, and ovaries or testis were positively correlated with the expression levels of GnRH in the hypothalamus.

The high degree of identity between the striped hamster PRLR sequence and those of the golden hamster, *Mus musculus*, and *Rattus norvegicus* is illustrated in Supplementary Figure S1. The deduced signal peptide of PRLR in the striped hamster and the golden hamster contain 23 amino acid residues, while that of *Mus musculus* and *Rattus norvegicus* have 19 amino acid residues. Twelve cysteines were detected in the PRLR of the striped hamster, the golden hamster, and *Mus musculus*, but only eleven cysteines were detected in the *Rattus norvegicus*. That is because the nucleotide of *Mus musculus* PRLR at the ORF 1765 T was substituted by the nucleotide C, and the code TGC for cysteine was transferred into CGC code for arginine. Five potential Asn-linked glycosylation sites were detected in the striped hamster and the golden hamster, while four sites were detected in the *Mus musculus* and *Rattus norvegicus*, as 1022 A was substituted by G, and the code AAT for asparagine was changed to AGT for serine. This nucleotide substitution may lead to some differences in regulating the structure and activity of PRLR. Two prolactin-binding motifs at the extracellular domain of PRLR were all found in the striped hamster, the golden hamster, *Mus musculus*, and *Rattus norvegicus*. The PRLR transmembrane region in the striped hamster also had high similarity with that of the golden hamster, *Mus musculus*, and *Rattus norvegicus*. The high similarity and some differences further demonstrated that the PRLR was conservative and divergent among various breeds. Two repeated units in the extracellular domain of the PRLR were also detected, which was similar to the chicken (24), the pigeon (25), and the turkey (12). The specific biological function of the repeated units remains to be identified, but each repeated unit contained four paired cysteine residues and a conserved ligand binding motif, which indicated that each PRLR may simultaneously interact with two PRL molecules.

The expression profile of PRLR in the striped hamster is developmental stage-dependent. *PRLR* gene may play an important role in the attainment of sexual maturity (4), which is consistent with our results. In our study, the PRLR mRNA in the hypothalamus, pituitary, and ovaries or testis of the 7-week-old animals showed higher levels, which may promote the sexual maturity. PRLR may regulate the development of ovaries (3), and the transcription of PRLR in the ovaries is complicated and regulated by developmental stages and hormones (26). PRLR mRNA was also detected in the ovaries of the striped hamster, which indicated an important regulating factor for ovarian development.

The expression profile of PRLR in the striped hamster was tissue-dependent. PRLR was expressed in an extensive range of tissues, but the PRLR mRNA level varied among different tissues. The expression profile of PRLR in the striped hamster was also sex-dependent. A sex difference exists in pituitary and hypothalamic PRLR mRNA in *Gallus domesticus*, with lower levels in both tissues in females than that in males (27), which is consistent with our results in the pituitary and hypothalamus of 13-week-old and 1.5-year-old female and male striped hamsters. The PRLR mRNA in the pituitary and hypothalamus of 7-week-old striped hamsters had no significant sex difference.

Haplotypes of PRLR in chickens are significantly associated with egg production (4), which indicated an important role in reproduction; our results are consistent with this. The PRLR genotype is associated with the number of live sperm in boars (28) and the number of total offspring and live offspring in pigs (29). The secretion level of prolactin varies with the estrous cycle, and a secretion surge of prolactin occurs in the pre-ovulatory phase (30), which means that PRL is an important regulating factor in ovulation.

In our study, significant correlations of PRLR mRNA in the hypothalamus, pituitary, and ovaries or testis with the expression levels of GnRH in the hypothalamus of the striped hamster showed that PRLR also had an important role in the reproduction of the striped hamster.

From our study, we concluded that the PRLR sequence in the striped hamster had a common and specific character compared with the PRLR sequence in other animals, and the expression profile of PRLR in the striped hamster was developmental-, sex- and tissue-dependent. PRLR plays an important role in gonad development and reproduction process, and its specific mechanism and effect need further study.

## Supplementary Material

Click here to view [pdf].
